# Feel like you belong: on the bidirectional link between emotional fit and group identification in task groups

**DOI:** 10.3389/fpsyg.2015.01106

**Published:** 2015-08-04

**Authors:** Ellen Delvaux, Loes Meeussen, Batja Mesquita

**Affiliations:** Department of Psychology, Center for Social and Cultural Psychology, University of Leuven, Leuven, Belgium

**Keywords:** emotions, emotional fit, group identification, small group dynamics, longitudinal, structural equation modeling, multilevel models, path analysis

## Abstract

Three studies investigated the association between members’ group identification and the emotional fit with their group. In the first study, a cross-sectional study in a large organization, we replicated earlier research by showing that group identification and emotional fit are positively associated, using a broader range of emotions and using profile correlations to measure group members’ emotional fit. In addition, in two longitudinal studies, where groups of students were followed at several time points during their collaboration on a project, we tested the directionality of the relationship between group identification and emotional fit. The results showed a bidirectional, positive link between group identification and emotional fit, such that group identification and emotional fit either mutually reinforce or mutually dampen each other over time. We discuss how these findings increase insights in group functioning and how they may be used to change group processes for better or worse.

## Introduction

Partners of couples and other dyads who spend time together, and members of small groups, such as work and sport teams show higher emotional similarity than what would be expected by chance (George, 1990; Totterdell et al., 1998; Bartel and Saavedra, 2000; Totterdell, 2000; Barsade, 2002; Anderson et al., 2003; Gonzaga et al., 2007; Ilies et al., 2007; Tanghe et al., 2010). This similarity, or “emotional fit,” may point to a shared perspective: emotions are appraisals of the (social) context, and emotional fit points to some sharing of appraisal (e.g., Parkinson, 1996; Fischer and Manstead, 2008; Mesquita et al., 2012; Elfenbein, 2014). For instance, anger signals that a person is unhappy with a situation, for which someone else is held responsible; in expressing anger, a person feels powerful and in control of the situation (Frijda et al., 1989; Kuppens et al., 2003). When two colleagues are angry because another colleague showed up late for a meeting, they interpret the situation similarly. Therefore, emotional fit stands for an alignment of interaction partners.

Individuals who are invested in their relationships and groups appear to have higher emotional fit. Indeed, in small group research, high identifiers’ emotions were more related to the group average than were the emotions of low identifiers (Totterdell et al., 1998; Totterdell, 2000; Tanghe et al., 2010). One possible explanation is that high identifiers are more receptive to other group members’ take on reality, and thus more readily adopt their emotions. This has been the most common interpretation of the link between group identification and emotional fit with the group; it suggests directionality *from* group identification *to* emotional fit. However, most of the evidence merely establishes a correlation between group identification and emotional fit, without showing directionality of the process.

An alternative view would be that group identification is an outcome of emotional fit. In this case, experiencing emotions that are typical of the group would be the very reason to feel belonging. If emotions reflect people’s position in the (social) world, then it would make sense that emotional similarity is an important ground for association with a group. For instance, feeling identified with Democrats may be largely based on feeling like a Democrat (Smith et al., 2007). It is plausible that individuals whose emotions resemble those of the majority of a group (In the 2008 elections, the Democrats were “hopeful,” “energized,” and “spirited,” among others; Dance et al., 2009) may become attracted to this group, and attach greater importance to it; over time, this would lead to stronger connections with the other group members. Although previous research did establish the *association* between emotional fit and group identification, there is no research to date that documents how group identification *follows* emotional fit. In the current research, we go beyond the association between group identification and emotional fit, and investigate their mutual influence over time.

### From Group Identification to Emotional Fit

Several researchers have suggested that group identification precedes emotional fit (Totterdell et al., 1998; Totterdell, 2000; Tanghe et al., 2010). There are two putative pathways (Haslam, 2004; Haslam et al., 2011). First, high identifiers pay more attention to other group members, because the group is very central to their identity. Therefore, high identifiers more readily pick up on (emotional) cues sent by other group members, and more readily adjust. Second, high identifiers who embody the group’s values, goals, and possibly emotions, serve as models to the other group members. In this case, other members adjust their emotions to fit those of the high identifiers. Regardless of the pathway, the result would be that members’ group identification predicts their emotional fit.

Evidence from correlational studies is consistent with this prediction, but does not address the direction of the link. For instance, in research with teams of nurses, accountants, or cricket players, commitment to the team predicted stronger emotional fit (Totterdell et al., 1998; Totterdell, 2000). Similarly, in teams of service employees, the emotions of high team identifiers were more closely linked to the emotions of their team than the emotions of low team identifiers (Tanghe et al., 2010). Furthermore, among self-identified Republicans and Democrats in the US, the emotions of those who were highly identified resembled the average emotional profile more than the emotions of those who were less identified (Smith et al., 2007).

To our knowledge, the only study that tested the direction of the link between group identification and emotional fit was done by Tanghe et al. (2010; Study 2). In this study, the authors manipulated both group identification and the group’s emotions at the same time, and found that high identifiers adjusted their emotions more toward the emotions of the group than low identifiers. Although this study demonstrated a causal link from group identification to emotional fit, it suffered from limitations. First, group membership was only imaginary: the participants imagined being part of a team. Second, the manipulation of identification rather than identification *per se* may have introduced emotional fit: as part of the identification-manipulation, participants had to imagine that “they fit well with the team” (Tanghe et al., 2010, p. 349) and that “there was a good match between themselves and the other team members” (p. 349).

In sum, despite its theoretical appeal, evidence that group identification promotes emotional fit is limited. In the current research, we investigate the causal link from group identification to emotional fit longitudinally.

### From Emotional Fit to Group Identification

Emotions may also be “the glue that sticks group members together” (Barsade and Gibson, 1998). Emotional fit itself may strengthen an individual’s felt connection to the group, thus amounting to higher group identification. In this case, group identification would be an outcome rather than a precursor of emotional fit. Consistent with this idea, one study found that ingroup identification increased after individuals were either made happy about the ingroup or angry toward an outgroup (Kessler and Hollbach, 2005). Similarly, one’s perceived fit with the emotions of other ingroup members led to stronger identification with the ingroup (Livingstone et al., 2011). These results suggest that group identification may be the result of emotional similarity between ingroup members, rather than merely its antecedent.

The findings on emotional fit are corroborated by a larger body of research showing that fit in other domains contributes to group members’ identification. For instance, a meta-analysis on person-organization fit and work attitudes showed that employees’ objective fit with the values, goals and personality characteristics that were central to their organization predicted their commitment to the organization (Verquer et al., 2003). Similarly, members’ value fit predicted their identification with the group several weeks later (Meeussen et al., 2014). Finally, when members of minimal groups communicated their ideas about a subsequent negotiation, inducing shared cognition among group members, their group identification had increased at the end of the negotiation (Swaab et al., 2007). It is possible, therefore, that the relationship between emotional fit and group identification goes in the other direction, with fit leading to increased group identification. The current research investigates the causal link from emotional fit to group identification longitudinally.

### The Current Research

In three field studies, we aimed to replicate and extend existing research on the association between group identification and emotional fit. The first study was meant to replicate the results from earlier cross-sectional research, using improved methods. The next two studies followed the direction of the relationship between group identification and emotional fit longitudinally, and tested whether either link is stronger than the other.

## Study 1

The aim of the first study, a cross-sectional study with different teams of a large organization, was to replicate the positive association between group identification and emotional fit with the group found by earlier research (Totterdell et al., 1998; Totterdell, 2000; Tanghe et al., 2010) with stronger measures of emotions and emotional fit.

### Method

#### Participants

Participants were 789 employees of a large, semi-governmental Belgian organization who were members of 85 teams, each consisting of 4–33 members (*M* = 9.28, SD = 4.91). Of the 789 participants, 491 were men (62%)^1^. Participants were on average 43.5 years old (SD = 9.73). They had been employed by the same organization for an average of 18 years (SD = 11.67), and had joined their current team for an average of 9.8 years (SD = 8.88). Since participants included both French-speaking and Dutch-speaking Belgians, the questionnaires were administered in French and Dutch respectively^2^. Participants completed the questionnaire in the language of their choice: 46% of the participants chose the French version (*n* = 362), and 54% the Dutch (*n* = 427).

#### Procedure

The current study was part of a larger research on “Diversity at the workplace” that took place in the organization. After the director of the organization had given his consent, we selected teams to be included in our study. Out of a list of all available teams, we selected the 122 that consisted of 25 employees or less^3^, because we assumed that the employees of these teams would interact with each other. Team leaders of all 122 potential teams were contacted by phone; after several attempts, 15% of the team leaders (*n* = 18) could not be reached, and 2% (*n* = 2) declined. Of the remaining teams (*n* = 102), 84% (*n* = 85) participated in the study.

The team leaders received the number of French and Dutch questionnaires needed for their teams, and distributed them among the members. Employees who consented to partake in the study, completed the questionnaire individually during work hours; filling out the questionnaire took approximately 30 min. After completing the questionnaire, employees returned their completed questionnaires in a sealed envelope to their team leader. The team leaders collected the envelopes from all team members, and mailed them back to the researchers.

#### Measures

***Team identification***

Team identification was measured by a seven item-scale (Cronbach’s alpha = 0.73): four items were taken from the identification-scale by Ellemers et al. (1999) (e.g., “I identify with the other members of my team”), and three items from the identification-scale by Roccas et al. (2008) (e.g., “Other teams can learn a lot from our team.”). Participants rated their agreement with each of the items on 5-point Likert scales ranging from 1 = *Totally disagree* to 5 = *Totally agree*. The mean rating of team identification across participants was 3.54 (SD = 0.65).

***Emotional fit***

Participants rated to what extent they experienced each of 24 emotions during the last month, when they worked together with the other members of their team. We expanded the commonly used list of affect items (e.g., nervous, enthusiastic; eight items; e.g., Totterdell et al., 1998; Totterdell, 2000; Tanghe et al., 2010), and added 16 emotion items to more fully reflect the emotion domain (Barrett et al., 2007; e.g., respect for my colleagues, ashamed of my group). Participants rated all emotion items on 5-point Likert scales ranging from 1 = *Very weak* to 5 = *Very strong*.

To measure participants’ emotional fit with their team, we used profile correlations (cf. De Leersnyder et al., 2011). Profile correlations measure the co-occurrence of a range of emotions. They provide an objective measure of fit, since the patterns of individual group members and their group are based on different sources (Kristof-Brown et al., 2005). To calculate profile correlations, we correlated participants’ own emotional pattern (across 24 emotions) with the average emotional pattern of their team, excluding their own values from this team average. Profile correlations have the advantage over difference measures that (a) they take into account information from a whole range of emotions, rather than averaging across these emotions, and (b) they take into account individual differences in scale use. The correlations ranged between –1 and 1, indicating the emotional fit with the team. Participants’ emotional fit was calculated if they responded to at least 19 out of 24 emotions. Because the measure of emotional fit was skewed (more data points when getting closer to 1), we transformed the correlations into Fisher’s *z* scores before conducting the remaining analyses (Fisher, 1921). The mean emotional fit across participants (Fisher’s *z* score) was 1.05 (SD = 0.56).

#### Analyses

We specified a two-level random intercept model, reflecting the nested nature of the data (employees within teams; Hox, 2002). Our main independent variable, team identification, and most control variables (gender, age, team tenure, leadership, and language) were situated at the individual level; the control variable team size was a team-level variable.

### Results

As expected, we found a positive relationship between team identification and emotional fit with the team: team identification predicted fit to the average emotional pattern of the team (β = 0.40, SE = 0.03, *p* < 0.001)^4^ (see Table 1).

**TABLE 1:** **Team identification predicts emotional fit in Study 1**.

	**Emotional fit**			
	**Regression weight**	**Standard error**	***t*-test (df)**	***p*-value**
Intercept	**0.874**	**0.066**	**13.22 (94.66)**	**<0.001**
Gender	0.029	0.041	0.70 (694.12)	0.487
Age	0.000	0.002	0.07 (694.95)	0.941
Team tenure	–0.003	0.002	–1.27 (691.15)	0.201
Leadership	**0.091**	**0.041**	**2.21 (651.09)**	**0.027**
Language	–0.036	0.042	–0.85 (521.56)	0.394
Team size	**0.013**	**0.005**	**2.42 (53.29)**	**0.019**
Group identification	**0.400**	**0.027**	**14.68 (687.96)**	**<0.001**

Bold numbers represent significant effects.

With regard to the control variables, there was a main effect for leadership (β = 0.09, SE = 0.04, *p* = 0.03): leaders showed more emotional fit with their team than the other employees. Furthermore, team size emerged as a significant predictor (β = 0.01, SE = 0.005, *p* = 0.02): employees of larger teams showed relatively more emotional fit.

### Discussion

Earlier research showed that compared to less identified team members, the affective state of strongly identified team members is more closely linked to the average affect in the team (Totterdell et al., 1998; Totterdell, 2000; Tanghe et al., 2010). In our research, we similarly found that members’ team identification was positively associated with their emotional fit to the team. Team members reported on emotions they had experienced during the last month, when spending time with the team. The emotion scales were selected to cover the full emotion domain, and are more representative of the experiences of team members than measures of positive or negative affect only. Moreover, the correlational measure of emotional fit used in this research does not suffer from the same disadvantages that are associated with the difference measures used in previous research.

The results held true when controlling for gender, age, team tenure, leadership, language, and team size. The control variable leadership itself showed an interesting and intuitive relationship with emotional fit, and suggests that indeed group leaders’ emotions shape the emotions of their followers (e.g., Sy et al., 2005; Haslam et al., 2011). Team size too predicted emotional fit, but this may have been a methodological artifact: the average group pattern in larger teams is based on more observations and is thus less likely to be “extreme.”

## Study 2

The aim of the second study was to establish the link between group identification and emotional fit longitudinally and to test the directionality of this effect. More specifically, we tested if members’ group identification at one point in time predicts their emotional fit with the group at the next, controlling for their emotional fit with the group at the previous point in time. We also tested if members’ emotional fit at one point in time strengthens their group identification at the next, when controlling for their group identification at the previous point in time.

### Method

#### Participants

We followed 68 task groups, each consisting of four to six second-year psychology students (*M* = 4.93, SD = 0.31) at a Dutch-speaking university in Belgium, throughout their collaboration on a joint project. Participants received an online questionnaire (in Dutch) at four different times; all students (*N* = 295) completed the questionnaire at least once during this collaboration. Attrition rates were low: 83% of the participants completed all questionnaires, and the rate of participation ranged from 98% in the first wave to 88% in the fourth. Because there were no differences between participants who did and did not complete all questionnaires, we included all participants in the analyses [Little’s (1988) Missing Completely at Random-test, *χ*^2^(168) = 158.90, *ns*].

The majority of the participants were female (88%); on average, participants were 20.39 years old (SD = 1.20). Demographics reflect the population characteristics of the student body (i.e., second-year psychology students). Participants received 10€ when they completed all four questionnaires, and 3€ when they completed any number lower than four.

#### Procedure

The study was approved by the Ethics Board of the University of Leuven, and participants signed an informed consent to agree to participate in the study. We recruited all students from a sophomore methods course for psychology majors. The course took a full semester (13 weeks), in which students conducted research. Measurement points marked the end of different steps in the research process: (1) completion of a literature review (week 2), (2) formulating hypotheses (week 4), (3) collecting and analyzing data (week 10), and (4) writing a research report (week 13).

The collaborative project was personally important for the students, because it was worth a full semester credit. Students reported working on the project for an average of 4.36 h per week (SD = 2.37); about one third of this time, they collaborated with the whole group on the project (*M* = 1.45 h, SD = 1.25). Good collaboration paid off, since 90% of the final course grade was based on the group’s performance.

#### Measures

***Group identification***

Group identification was measured with six of the seven items used in Study 1; the item “I feel strongly connected to the members of my team” was accidentally omitted from this scale. Participants rated their agreement with each of the items on 5-point Likert scales ranging from 1 = *Totally disagree* to 5 = *Totally agree*.

***Emotional fit***

Participants rated to what extent they had felt each of 14 emotions (e.g., pride about the group, angry at the other group members) when working together with the other members of their group in the time since the last measurement. To limit the burden on participants (who were asked to fill out the questionnaire at four different times), we reduced the number of emotions in this study as compared to Study 1. Participants rated the emotion items on 5-point Likert scales ranging from 1 = *Very weak* to 5 = *Very strong*. Emotional fit was calculated using the procedure as described for Study 1.

Table 2 summarizes the means, standard deviations and Cronbach’s alphas for group identification and emotional fit at each of the four waves.

**TABLE 2:** **Means, standard deviations and reliabilities for group identification and emotional fit with the group (Study 2)**.

	**Week 2**	**Week 4**	**Week 10**	**Week 13**
	***M*(SD) Cronbach’s alpha**			
Group identification	3.59 (0.60) 0.77	3.56 (0.62) 0.78	3.51 (0.74) 0.86	3.40 (0.77) 0.85
Emotional fit (Fisher z-transformed)	1.10 (0.63)	1.11 (0.64)	1.00 (0.58)	1.11 (0.63)

#### Analyses

To investigate the interplay between group identification and emotional fit, we used multilevel structural equation modeling. More specifically, we estimated a fully cross-lagged path model. We were particularly interested in the cross-lagged paths because they estimate the effect of one variable at one time point on another variable at the next time point, controlling for the other variable at the previous time point as well as controlling for within-time associations. Hence, we were able to estimate the effect of group identification on emotional fit as well as the effect of emotional fit on group identification over time. We estimated all within-time correlations and autoregressive paths to control for their effects. Since individual group members were nested within task groups, we specified multilevel models to take into account that observations were not independent (Hox, 2002).

***Model specifications***

In structural equation modeling, model selection takes place by comparing the respective fit of different models. More restricted (= more simple) models are compared against less restricted (= more complex) models. The more restricted model is chosen over the less restricted model when model restrictions do not result in significant decreases of the model fit.

In our data, we first tested an unrestricted model with all parameters freely estimated. Next, we compared this model with two restricted models. First, we did not predict that the links between emotional fit and group identification in whichever direction would differ across time. Therefore, we restricted the cross-lagged paths to be equal over time and compared the restricted model to the unrestricted model. If the model fit does not significantly decrease by equating these paths, the effects can be considered to be equal over time.

Second, we also did not have any *a priori* ideas about the direction of the link between group identification and emotional fit. To test whether the effect of emotional fit on group identification was stronger than the effect of group identification on emotional fit, or *vice versa*, we restricted the model by equating the links in both directions, and comparing this restricted model to the previous restricted model. If the model fit significantly decreases by equating these paths, one of the effects can be considered stronger than the other; if the model fit does not significantly decrease by equating these paths, the effects can be considered equally strong in both directions.

Each tested model’s fit was evaluated using two common indices to evaluate model fit: a model with an RMSEA-value than 0.10, and preferably 0.06, and a CFI-value higher than 0.90, and preferably 0.95 indicate adequate to excellent model fit (Hu and Bentler, 1999; Kline, 2005). The same indices are also used to evaluate change in model fit when testing a more restricted model against a less restricted model. When the change in RMSEA is smaller than 0.015 and the change in CFI is smaller than –0.01, the more restricted model is chosen over the less restricted model (Vandenberg and Lance, 2000; Cheung and Rensvold, 2002).

### Results

**FIGURE 1:**
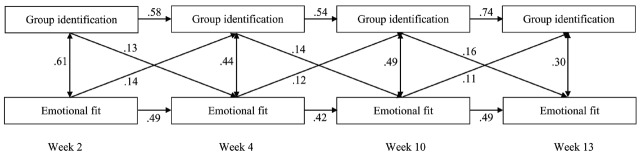
**Fully cross-lagged multilevel model outlining the relation between group identification and emotional fit over time (Study 2).** The numbers in the figure represent the standardized betas. Model fit was excellent: CFI = 0.99, RMSEA = 0.05. The results converged with and without controls (age, gender and number of friends at the start of the project), as well as with the full identification scale, and the identification subscales (Ellemers et al., 1999 vs. Roccas et al., 2008). The presented model is the model without controls and with the full identification scale. All *p*’s < 0.001.

The selected model (see Figure 1) shows a mutual, positive relationship between members’ group identification and their emotional fit over time. Equating the cross-temporal paths from group identification to emotional fit as well as from emotional fit to group identification, did not result in a significant decrease of the model fit (ΔRMSEA = –0.015, ΔCFI = 0.004), suggesting similar effects over time. More specifically, members’ group identification at one point in time predicted their emotional fit with the group at the next, controlling for their group identification at the previous time. Conversely, members’ emotional fit with their group at one time predicted their group identification at the next, controlling for their own emotional fit at the previous time. Thus, the higher (lower) group members’ identification at one point in time, the higher (lower) their emotional fit at the next, and *vice versa*.

Moreover, it was possible to equate the path from group identification to emotional fit to the path from emotional fit to group identification (ΔRMSEA = –0.004, ΔCFI = 0.001), indicating that the effect was equally strong in both directions. These effects were found, both when controlling for within-time correlations between group identification and emotional fit, and when controlling for the autoregressive effects of group identification and emotional fit; all within-time correlations and autoregressive effects were significant.

In sum, our results document a bidirectional effect that was equally strong in both directions: not only did members’ group identification predict their emotional fit with the group, but members’ emotional fit with the group also predicted their group identification.

### Discussion

In Study 2, we extended the results of Study 1 by testing the directionality of the link between members’ group identification and their emotional fit to the group. A multilevel cross-lagged path analysis showed that group identification and emotional fit with the group mutually influenced each other over time. Moreover, these effects were equally strong in both directions. To our knowledge, our study is the first study that provides evidence for a bidirectional link between group identification and emotional fit using a longitudinal design.

## Study 3

The third study aimed to replicate the results of Study 2, using sociometric data to measure members’ identification to their group. Sociometric data provide an implicit measure of group identification. They allow to infer a person’s strength of their connection to the group from their connections with every individual group member. Convergence of the results based on this implicit measure of identification with those based on the explicit measures of group identification used in the previous studies would inspire confidence in the conclusions.

We followed student work groups over time, and measured group identification by examining the strength of the connections between different group members. Whereas the student work groups in Study 2 consisted of white and primarily female psychology students, the work groups in Study 3 consisted of ethnically diverse and primarily male engineering students. As in Study 2, we investigated the mutual influence between group identification and emotional fit with the group over time.

### Method

#### Participants

We followed 33 task groups throughout their collaboration on a project; each task group consisted of five to seven group members plus one group leader (group size: *M* = 7.24, SD = 0.66). The group members were first-year engineering students and the group leaders were fourth-year engineering students at a French-speaking university in Belgium. All students (*N* = 239) completed a paper-and-pencil questionnaire (in French; see text footnote 2) at least once during their collaboration. Attrition rates were somewhat higher than in Study 2, but still 72% of the participants completed all three waves. The participation rate was 90% in wave 1, 79% in wave 2, and 92% in wave 3. We included all participants in the analyses, since participants with and without missing data did not significantly differ from each other on the variables of interest [Little’s (1988) Missing Completely at Random-test, *χ*^2^(85) = 93.69, *ns*].

On average, group members were 18.5 years old (SD = 1.12) and group leaders were 22 years old (SD = 1.98); 79% of the group members were men and 70% of the group leaders were men. Demographics reflect the population characteristics of the student body (i.e., first- and fourth-year engineering students). All participants took part in the study voluntarily. Students of two participating groups received cinema tickets via a lottery after the study was completed.

#### Procedure

The study was approved by the Ethics Board of the University of Leuven, and participants signed an informed consent to agree to participate in the study. We recruited participants during the launch session of an engineering course. For this course, students worked together on a group project for 6 months. During this period, they designed and built a technical device that could heat water by means of physical activity (e.g., pedaling or rowing); the group leader guided the process. At the end of the project, students handed in a written report on their group project and presented their prototype to an external jury. The group project was significant, both in terms of its place in the curriculum and in terms of time spent on it. On average, the students reported working on the project on average 4.73 h a week with the whole group (SD = 3.96) and 4.67 h by themselves (SD = 4.18). At three times during the project, students completed the questionnaire: in week 7, week 21 (with six weeks of holiday and exams in between) and in week 24 (after presenting their prototype to an external jury).^5^

#### Measures

***Group identification***

In the current study, we measured group identification by sociometric data rather than by a self-reported summary statement. Group identification was the average strength of the ties of an individual group member to all the other group members. Ties were measured as the extent of (1) liking, (2) getting along with, and (3) being attuned to a particular group member. To obtain a group member’s identification, we first averaged an individual participant’s ratings (of all the other group members) per item; we then averaged across the three items. Averaging across items was justified, as factor analyses yielded one single factor at each point in time, and the reliabilities of the resulting three-item scales were excellent (see Table 3).

**TABLE 3:** **Means, standard deviations and reliabilities for group identification and emotional fit with the group (Study 3)**.

	***M* (SD) Cronbach’s alpha**		
	**Week 7**	**Week 21**	**Week 24**
Group identification	3.68 (0.54) 0.84	3.71 (0.53) 0.92	3.85 (0.55) 0.92
Emotional fit (Fisher z-transformed)	1.10 (0.44)	1.10 (0.48)	1.20 (0.48)

***Emotional fit***

Emotional fit was measured in the same way as in Studies 1 and 2. In this study, the emotional concordance score was based on 27 emotions (e.g., respect toward other group members, enthusiastic, irritation toward other group members, nervous). Participants’ emotional fit was calculated if they responded to at least 22 out of 27 emotions.

Table 3 summarizes the means, standard deviations and Cronbach’s alphas for group identification and emotional fit.

#### Analyses

The analytic strategy in this study is the same as in Study 2. To investigate the relationship between group identification and emotional fit with the group, we estimated multilevel cross-lagged models using structural equation modeling techniques.

### Results

**FIGURE 2:**
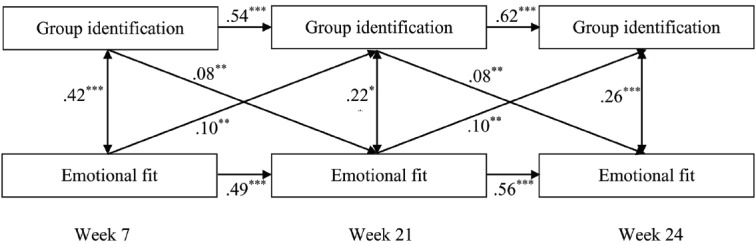
**Fully cross-lagged multilevel model outlining the relation between group identification and emotional fit over time (Study 3).** The numbers in the figure represent the standardized betas. Model fit was good: CFI = 0.97, RMSEA = 0.07. The results converged with and without controls (age, gender and leadership). The presented model is the model without controls. *p < 0.05, **p < 0.01, ***p < 0.001.

Figure 2 shows support for a bidirectional link between group identification and emotional fit. As expected, we found a mutual, positive relationship between members’ group identification and their emotional fit over time. Given that the decrease in model fit was not significant, we equated the paths from group identification to emotional fit and from emotional fit to group identification over time (ΔRMSEA = –0.002, ΔCFI = –0.007), such that their effects were equal over time. More specifically, members’ group identification at one point in time predicted their emotional fit with the group at the next, controlling for their group identification at the previous time. Similarly, members’ emotional fit with their group at one time predicted their group identification at the next, controlling for their own emotional fit at the previous time.

Moreover, the model fit did not deteriorate significantly when the paths from group identification to emotional fit and from emotional fit to group identification were set equal to each other (ΔRMSEA = –0.004, ΔCFI = –0.002). Thus, the link is bidirectional and equally strong in both directions. As in Study 2, the results were true when controlling for within-time correlations between group identification and emotional fit as well as when controlling for the autoregressive effects of both group identification and emotional fit; all of these effects were also significant.

### Discussion

In Study 3, we replicated the results of Study 2: we found evidence for a bidirectional link between group identification and emotional fit over time. The results of Study 2 and 3 converged despite important differences between the two studies. A first difference concerns the measurement of group identification: In study 2, we measured self-reported connectedness with the group, and in Study 3, we used sociometric data. Second, sample characteristics differed: Groups in Study 2 consisted of White and majority female students, whereas the groups in Study 3 were ethnically diverse, and predominantly male. Moreover, the participants of Study 2 were psychology students, whereas the participants of Study 3 were engineering students. Finally, the questionnaires in Study 2 were administered in Dutch, whereas the questionnaires in Study 3 were administered in French. In sum, two longitudinal studies with naturally occurring student work groups yielded converging evidence for a bidirectional link between group identification and emotional fit with the group.

## General Discussion

Across three studies, following “real” interactive task groups working on tasks that were meaningful and important to them, we found that group identification predicts members’ emotional fit, a link that had been suggested by previous research (Totterdell et al., 1998; Totterdell, 2000; Tanghe et al., 2010). We replicated this relationship, using different measures of group identification and considering a broader range of emotions than had been included by these previous studies. Moreover, we used profile correlations to measure emotional fit instead of difference measures. Emotional fit in terms of profile correlations points to an alignment of group members’ perspective on the situation, and this alignment may be initialized by a strong connection with the group. In addition, we also found evidence for the reverse effect: emotional fit also predicts group identification. When group members’ emotions are aligned, and thus their perspectives on the situation as well, they start feeling more connected with their group.

Our two longitudinal studies established a bidirectional relationship between group identification and emotional fit. Furthermore, the effects were equally strong in both directions: there were feedback loops between group identification and emotional fit, such that group identification and emotional fit either mutually reinforce or mutually dampen each other. In the context of small, interactive task groups, both group identification and emotional fit are thus dynamic rather than stable over time.

### Relevance for Group Outcomes

The dynamic interplay between group identification and emotional fit suggests that a change in the one variable, brings about a change in the other. This may set in motion a positive or negative spiral, affecting members’ well-being, motivation and performance in the group. Indeed, higher levels of emotional fit and group identification have both been associated with positive outcomes.

On the one hand, many studies have shown that emotional fit benefits relationship outcomes. For instance, emotional fit in dyadic relationships predicts satisfaction with the relationship (Locke and Horowitz, 1990; Anderson et al., 2003; Gonzaga et al., 2007; Verhofstadt et al., 2008; Townsend et al., 2014). Similarly, emotional fit with one’s culture is positively associated with relational well-being (De Leersnyder et al., 2014). Finally, in top management teams, members’ affective fit with the team is positively related to their satisfaction with the interpersonal relationships in the team (Barsade et al., 2000).

On the other hand, group identification has been found to motivate members to contribute to the group’s goals. Members who are highly identified with the group are thus more motivated to work on the group’s tasks as well as to perform better on these tasks (Worchel et al., 1998; Ellemers et al., 2004; Meeussen et al., 2014).

### Practical Implications

Team members who are aware of the dynamic interplay between group identification and emotional fit, will be able to break a negative spiral or promote a positive spiral. Similarly, group interventions leveling at improving either group identification or emotional fit may promote positive outcomes for the group (e.g., Anderson et al., 2003; Ellemers et al., 2004). Team building activities provide a good framework to attain these goals.

On the one hand, team building activities may strengthen group identification. Previous research has shown that group members identify more strongly with their group after personally contributing to their group’s identity (Swaab et al., 2008; Jans et al., 2012; Meeussen et al., 2014). An intervention including all group members, aiming at jointly building a group identity (cf. “an inductive route to social identity formation,” Jans et al., 2012) would be one way to increase members’ group identification.

On the other hand, team building activities may be used to increase emotional fit by encouraging discussion about the meaning of events. For instance, we think of a framework like the one provided by Clark and Wilkes-Gibbs (1986), in which interaction partners took turns communicating about ambiguous stimuli until they reached a common understanding of the stimuli. During this process, and throughout different interaction turns, partners increasingly reached agreement on the meaning of stimuli. Similarly, exercises where group members discuss emotional scenarios with the aim of reaching a common understanding of the situation may improve the process of emotional appraisal, thus increasing emotional fit.

### Limitations and Future Directions

Although we have established the bidirectional link between group identification and emotional fit longitudinally, the processes underlying this link have yet to be explored. As discussed in the introduction, there are two different processes that may bring about high identifiers’ stronger emotional fit to the group. High identifiers may either more readily align their emotions with those of other group members, or they may set an example to the other group members (Haslam, 2004; Haslam et al., 2011). Future studies should examine the conditions under which each of these pathways occur.

Another limitation, at least for the longitudinal studies, is that they focused on newly formed groups. The studies thus pertain to identification and emotional fit of group members who have just started to collaborate. Future research may study whether the bidirectional link remains over time, or only exists during group formation. Longitudinal research on teams that have been in existence for some time (such as the teams included in the first study) should be expected to shed light on this issue.

Furthermore, the current research describes general processes of identification and fit across group members. Future research may disentangle the trajectories of different types of group members: individuals with either high or low group identification, or either high or low emotional fit. To establish different trajectories, and thus monitor fluctuations over time, research might benefit from measuring group identification and emotional fit at shorter intervals.

Another direction for future research is to study how different mean patterns of emotional experience influence the outcomes associated with emotional fit. Emotional fit may not always be functional or advantageous. For instance, fit to a pattern that is characterized by high levels of anger may be less beneficial than fit to a pattern that is characterized by high levels of group pride. Similarly, fit to a pattern that is characterized by intense anger and low levels of other emotions may be less beneficial than fit to a pattern that is characterized by equally intense anger that is accompanied with high levels of respect and sympathy. The former in each comparison may lead to worse group outcomes, whereas the latter may benefit group outcomes.

## Conclusion

To conclude, three studies with real-life, interactive task groups yield a bidirectional link between group identification and emotional fit with the group. Over time, group identification predicted emotional fit, but the reverse link was found as well. Interventions that improve either one may thus affect both processes. This may lead to better group relationships and better group performance. Conversely, a decrease in either group identification or emotional fit may lead to deteriorations in both, and thus negatively affect group outcomes.

### Conflict of Interest Statement

The authors declare that the research was conducted in the absence of any commercial or financial relationships that could be construed as a potential conflict of interest.

## Footnotes

1 One participant was dropped from the analyses because we had reasons to believe he faked his answers: (a) he reported to be sleeping at work, (b) he reported to have no colleagues, yet he also claimed responsibility for 80 employees, (c) he only used the extreme ends of the scales.

2 The questionnaire was translated and back-translated from Dutch into French by a bilingual translator and checked by two researchers speaking both languages.

3 Two of the teams in our final sample consisted of more than 25 team members, because they had grown since the team list was made.

4 We also conducted the analyses for the two subscales of group identification separately. The results replicated the results with the combined identification-scale. In addition, the subscales were less reliable than the combined scale [Cronbach’s alphas of 0.58 for the scale by Ellemers et al. (1999) and 0.64 for the scale by Roccas et al. (2008)]. Therefore, we only report the results for the combined scale.

5 We did not succeed in our aim to make the intervals between measurement times similar. The timing of the different waves (at 7, 21, and 24 weeks) was primarily motivated by pragmatic considerations. After 7 weeks, the group leaders had the opportunity to distribute the questionnaire to their group members for the first time. The large gap between the first wave (week 7) and the second (week 21) was due to a 6-weeks semester break. Finally, the last questionnaire was distributed to the group members immediately after they presented their work to an external jury (week 24), and therefore right before the group’s dissolution.
